# Early retirement intentions: the impact of employment biographies, work stress and health among a baby-boomer generation

**DOI:** 10.1007/s10433-022-00731-0

**Published:** 2022-09-30

**Authors:** Lisa Toczek, Hans Bosma, Richard Peter

**Affiliations:** 1 Department of Medical Sociology, Institute of the History, Philosophy and Ethics of Medicine, Faculty of Medicine, Ulm University, Parkstrasse 11, 89073 Ulm, Germany; 2 Department of Social Medicine, Care and Public Health Research Institute (CAPHRI), Faculty of Health, Medicine and Life Sciences, Maastricht University, P.O. Box 5800, 6202 AZ Maastricht, The Netherlands

**Keywords:** Self-rated health, Effort-reward imbalance, Employment histories, LidA study, Life course approach, Cluster analysis

## Abstract

In recent years, early retirement decisions have become more frequent in the European Union despite political efforts to prevent early retirement. This is a growing problem for the social security system. The study focuses on a life course approach using employment biographies and investigates the influence of work stress and health on early retirement intentions. Data of employees who were born in either 1959 or 1965 of the German cohort study on work, age, health and work participation are analysed (*n* = 3338). By linking survey and register data from 1993 to 2011, a sequence analysis is conducted to identify employment biographies. To analyse the relationship between the employment biographies and intended early retirement, a longitudinal path analysis is computed and includes work stress, measured through effort-reward imbalance, and self-rated health. The statistical analyses identify three adverse employment biographies, i.e. part-time work, episodes of unemployment or marginal employment. In addition, two favourable employment biographies are determined, characterised by full-time work and few episodes of unemployment. The results of the path analysis show that employment biographies with high work-related stress have early retirement intentions. Among adverse employment biographies, indirect effects of poor health on the association between work stress and early retirement intentions are found. Unexpectedly, among full-time workers, work stress is also associated with early retirement intentions with an additional mediation through health. The findings of this study highlight the importance of the life course perspective when analysing retirement decisions. In addition to health-promoting interventions in the labour market, effects of psychosocial factors should be focussed on in order to reduce early exits from the labour market.

**Supplementary Information:**

The online version contains supplementary material available at 10.1007/s10433-022-00731-0.

## Introduction

In recent years, statutory retirement age has been raised in many member states of the European Union (EU). This is due to a considerable burden on the pension schemes initiated by increasing life expectancy and ageing workforces. In particular, the retirement of large birth cohorts in the coming decades and thus the decline of the working population will put pressure on the social security systems (European Commission 2020; Cahalin 2009). Yet, political efforts like changes in the statutory retirement age have had little impact on the individual’s intention to retire early (Hofäcker 2015, Wahrendorf et al. 2013, European Commission 2020, Hedge et al. 2006). Moreover, although health still is the most important factor for retirement decisions, research showed that a considerable number of workers with poor health continue to stay in the labour market (Burr et al. 2013). Hence, retirement decisions seem to be the product of rather complex push and pull processes. Among these processes, individual motivation to retire or to stay employed seems to be a relevant factor that influences intentions to retire early (Hasselhorn et al. 2014). In the process of retirement decisions, intention to retire and actual retirement are closely intertwined, with intention being a good predictor of actual retirement (Harkonmäki 2007; Solem et al. 2016; Beehr 1986; Beehr and Bennett 2007). Therefore, to better understand the motivations of older workers that retain them in the labour market, it is important to investigate the determinants of early retirement intentions.

### Determinants of early retirement intentions 

Health is the most frequently analysed and most prominent predictor of retirement decisions. Previous research found associations between poor health and intended early retirement (Harkonmäki 2007; Elovainio et al. 2005; von Bonsdorff et al. 2010; Siegrist et al. 2007). In addition, another study demonstrated that people with better self-rated health preferred late to early retirement (Sousa-Ribeiro et al. 2021). However, some earlier studies could not discover a clear association between poor health and early retirement intentions (Schreurs et al. 2011; Du Prel et al. 2019). Moreover, research indicated that good health can also be related to early retirement decisions especially among people who can afford to retire (Pond et al. 2010). The heterogeneous results regarding the association between health and intention to retire early indicate the complexity of early exit from the labour market. Nonetheless, poor health remains an important legal determinant of early retirement. Therefore, factors associated with poor health should deserve special attention in the research on determinants of early retirement intentions.

Previous research has shown that health is influenced by characteristics of adverse employment, such as disadvantaged or discontinuous work. Earlier studies identified an association between labour market disadvantages—particular non-standard work, career interruptions and repeated unemployment periods—and negative health outcomes, such as overall poor health and psychological distress (Gal et al. 2008; Wahrendorf et al. 2018; van Aerden et al. 2016; Benach et al. 2014). Longitudinal findings of recent research identified associations between adverse employment histories and poor health later in life (Wahrendorf et al. 2021; Hoven et al. 2021). In addition, employment histories were found to be important predictors of retirement decisions. Regarding discontinuous employment, results of a recent study illustrated that periods of unemployment could be associated with an early retirement age (Murray et al. 2019). Research demonstrated that non-standard, low-paid work and discontinuous employment are strongly linked to job insecurity and economic distress (Green et al. 2001; Kalleberg 2009) and high job insecurity was found to increase the risk of early retirement (Lund and Villadsen 2005). However, previous research demonstrated inconsistent results (Fisher et al. 2016). Browne et al. (2019) found only insufficient evidence about job insecurity and the effect on retirement decisions. Nevertheless, considering a strong association between poor health and early retirement intentions, we expect adverse employment biographies to be associated with early retirement intentions.

The decision to retire is influenced by a number of push and pull factors (Feldman 1994; Shultz et al. 1998). Push factors are negative factors that induce workers into retirement, such as poor health. Pull factors are positive factors that attract workers to retire (Shultz et al. 1998). Work stress has been identified as a push factor when people want to leave a negative work environment (Wang 2007; Reeuwijk et al. 2013; Fisher et al. 2016). In previous research, high work stress, particularly effort-reward imbalance (ERI), was found to be associated with early retirement intentions (Siegrist et al. 2007; Wahrendorf et al. 2013; Fisher et al. 2016; Du Prel et al. 2019). However, Browne et al. (2019) argued that there is insufficient evidence about the influence of ERI on retirement decisions. Regarding the influence on health, findings of Siegrist (1996) showed that ERI can result in adverse health effects. Work stress is not only associated with poor health but also with precarious work. Previous research of Clarke et al. (2007) and Lewchuk et al. (2003) found higher stress levels among workers in precarious employment compared to those with standard working conditions. In addition, both studies found stress-related health issues for workers in precarious employment (Lewchuk et al. 2003; Clarke et al. 2007). However, associations between ERI and adverse employment histories were found to be heterogeneous (Hoven et al. 2020).

### Life course perspective

The model of Beehr (1986) described retirement as a process which occurs over a long period of time: beginning with preference to retire, followed by decision to retire (intention) and finally actual retirement. Moreover, retirement decisions can be influenced through previous events in the employment trajectory. Hence, retirement cannot be described as a discrete event, but as a process over a long period of time and research should consider this using life course perspectives (Beehr 1986; Loretto and Vickerstaff 2013; Fisher et al. 2016). The theory of cumulative advantages/disadvantages of Dannefer (2003) emphasises the importance of life course perspectives. It claims that advantages or disadvantages can accumulate over life and social inequalities in early life stages can affect later life (Dannefer 2003). Recent research showed that adversity during childhood and adulthood is associated with later employment histories (Hoven et al. 2018). Therefore, to investigate early retirement decisions, the present study uses the life course perspective by considering whole employment biographies.

In the past few years, changes in the labour market have led to higher rates of the so-called atypical or non-standard employment, which can be found in low-income, middle-income and developed countries (Kalleberg 2009; Benach et al. 2016). Given the variety of labour market challenges, it is useful to define non-standard employment from a country-specific perspective. The norm of standard employment in mainly high-income countries such as Germany was characterised as being both full-time and permanent, and also stable in income (Benach et al. 2010). However, the German labour market with formerly predominant full-time employment has become more diversified with more non-standard, atypical employment, which are associated with adverse working conditions creating job insecurities (Siegrist 2016; Kalleberg 2009). Therefore, the increase and manifestation of non-standard employment in the labour market highlight an important change (Kalleberg 2009). In this study, we characterise adverse employment careers by discontinuous and disadvantaged working trajectories. Discontinuous employment is defined by interruptions in the career such as unemployment periods. Disadvantaged working conditions are defined as low-pay work with high job insecurity. Low-pay work, i.e. part-time employment, can affect the career in different ways. On the one hand, part-time employment can provide flexible work arrangements and good work-life balance. On the other hand, part-time work involves fewer working hours, which in turn leads to lower wages compared to full-time jobs. In addition, part-time employees are less satisfied with their salary and tend to be disadvantaged regarding career opportunities (Broughton et al. 2016). A particular employment status in the part-time work structure, which is associated with disadvantaged working conditions, is called marginal employment. This is characterised as low-income employment with a wage threshold of 450 Euros per month or up to 5400 Euros per year (Federal Ministry of Labour and Social Affairs 2020). Considering the major financial disadvantages both part-time and marginal employment can be defined as adverse working conditions characterised by low wages, limited social insurance coverage and high job insecurity (Botsch 2015).

### Aim and hypotheses

Labour market disadvantages can deteriorate health and increase work stress and moreover accumulate over time leading to early retirement intentions of employees. To analyse determinants of early retirement intentions, in our study, a life course perspective is applied by distinguishing different employment biographies and considering adverse working conditions. Previous studies predominantly used cross-sectional rather than longitudinal approaches (Elovainio et al. 2005; Harkonmäki 2007; Siegrist et al. 2007; Wahrendorf et al. 2013; Hofäcker and Naumann 2015). Research that applied a life course perspective of employment is still sparse and recent studies that analysed working conditions over time used retrospective data, which include potential recall bias (Wahrendorf et al. 2018; Hoven et al. 2018, 2020, 2021). In this study, we intend to fill the research gaps with the following hypotheses:

#### H_1_

People with adverse employment biographies want to retire earlier.

#### H_2_

Among adverse employment biographies, work stress is associated with early retirement intentions.

#### H_3_

Health influences the relationship between work stress and intended early retirement.

## Materials and methods

### Data source

Data were obtained from the German cohort study on work, age, health and work participation, the lidA study, and included German employees born in 1959 or 1965 who required to pay social security contributions (reference date: 31 December 2009) (Hasselhorn et al. 2014). In 2011 (baseline, *t*_0_), survey data were conducted via computer-assisted personal interviews with 6585 respondents (response rate: 27.3%) (Schröder et al. 2013). The 3-year follow-up survey in 2014 (*t*_1_) comprised 4244 interviews (response rate: 75.7%) (Steinwede et al. 2015). With written consent, the survey data were individually linked to the register data of the Institute for Employment Research, the so-called IEB data (Integrated Employment Biographies). The information regarding employment biographies was extracted from the IEB data and the information regarding work stress and health was obtained from the survey data (details are provided in the following subchapter).

The IEB data were retrieved from the annual reports submitted by employers to the social security authority. This social security data provided information on respondents’ employment biographies from 1975 onward for West Germany and from 1993 for East Germany. The data included information about full-time work, part-time work, marginal employment and unemployment. Information about marginal employment and unemployment was only available from 1999 onward due to the composition of the IEB data. The data were available on a daily basis for each person and all time-varying information was coded to the day (Hasselhorn et al. 2014).

**Fig. 1:**
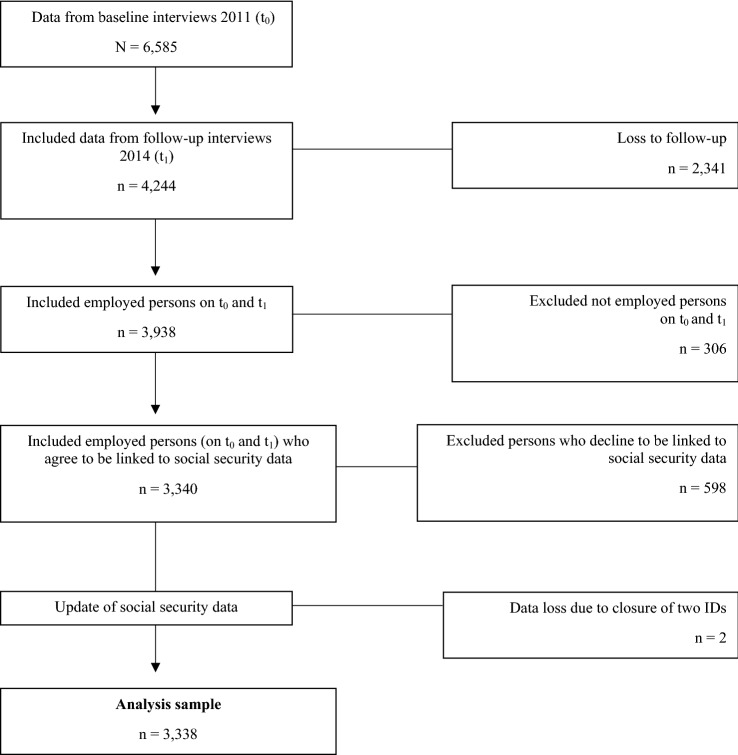
Decision tree for analysis sample

The final analysis sample included 3338 employees who were employed on the survey dates and gave written consent to linkage of the survey data with the register data (Fig. 1).

### Variables

#### Intention to retire early

Early retirement intentions were measured only at the follow-up survey (2014). Information was collected by asking about the desired retirement age: ‘Irrespective of the statutory retirement rules, up to what age would you like to work?’ with five categories: (1) 50–54, (2) 55–59, (3) 60–64, (4) 65–67, (5) beyond the statutory retirement age. Lower categories mark intentions to retire earlier.

#### Effort-reward imbalance (ERI)

Work stress was measured through the model of effort-reward imbalance (ERI). The ERI-questionnaire measures ‘effort’ and ‘reward’ with Likert-scaled items. The subscale ‘effort’ contains six items, the subscale ‘reward’ 11 questions. To measure ERI, a ratio is computed by dividing the subscale ‘effort’ by the subscale ‘reward’. In addition, a weighting factor of 6/11 is added to the denominator to adjust for the varying numbers of items in the subscales. Ratio values close to zero indicate low effort and high reward, in contrast to values above 1.0 which indicate high ERI imbalance and hence higher work stress (Siegrist et al. 2004). Therefore, higher values indicate an increase in work stress. ERI was measured by the survey data at both baseline and follow-up.

#### Self-rated health

Health was assessed by one self-rated question of the SF 12: ‘How would you rate your general health status?’ (Nübling et al. 2006). The response was given on a 5-point Likert scale: (1) very good, (2) good, (3) satisfactory, (4) not so good, (5) bad. Health was measured in the survey data at both baseline and follow-up. Higher values indicate poorer health.

#### Additional variables

Possible confounders were incorporated: sex, age, overcommitment, negative affectivity and indicators of socio-economic status. Sex was defined as (1) male, (2) female. Age was assessed through the birth cohorts of lidA, born in 1959 or 1965. As part of the ERI model, overcommitment was considered and relates to individual differences regarding how people experience the imbalance between effort and reward (Siegrist et al. 2004). Negative affectivity was included for potential reporting bias of the self-reported survey data. Indicators of socio-economic status were included. Education considered information regarding education and vocational training (Jöckel et al. 1998). The combined categories included (1) high, (2) intermediate and (3) low education (Supplementary Table 1). Occupational status was established in four categories: (1) professional, (2) middle management worker, (3) skilled worker and (4) unskilled worker. Information regarding income was determined as current net monthly income with categories from: (1) high, (2) middle high, (3) middle low and (4) low income. All information concerning the additional variables was taken from the baseline survey (2011).

### Statistical analysis

The statistical analysis was structured in two parts. First, sequence analysis was conducted to identify typical clusters of employment biographies and classified into adverse and favourable ones. Second, path analysis was performed to investigate the association between intended early retirement and both work stress and health in the generated employment biographies. To counteract possible bias, the final path analysis was adjusted for the confounders.

#### Sequence analysis

Information regarding the IEB data was utilised to identify the individual employment status for each year. This was available on a daily basis from employers’ yearly reports submitted to the social security authority. In preparation for a sequence analysis, the annual employment status of each individual was classified into one of six possible states: full-time work ‘F’, full-time and marginal work ‘FM’, part-time work ‘P’, marginal work ‘M’, unemployment ‘U’, no information ‘NI’. If individuals worked six months or longer per year in one employment status, they were assigned to that type. The status ‘no information’ contained individuals with either working less than six months in one employment status per year, or individuals with no social security contributions and therefore no information in the register data about their employment situation. After the classification, the sequence analysis was applied. The dissimilarities between sequences were measured using the optimal matching (OM) method. To transform one sequence to another, the distance between two sequences was calculated through costs: substitution costs and indel costs (insertions and deletions) (Abbott and Forrest 1986; Studer and Ritschard 2016). Substitution costs were calculated by means of observed transition rates (method ‘TRATE’). Indel costs were set constant at ‘1’, as recommended (Stegmann et al. 2013). Cluster analysis was conducted based on the calculated distance matrix with the Ward hierarchical clustering method and the OM technique. To define the appropriate number of clusters, graphical decision supports were used: dendrogram and elbow-criteria (Cornwell 2015; Stegmann et al. 2013).

To avoid systematic bias, the year 1993 was chosen as the starting point, because the register data do not contain any information about people born in East Germany before 1993. The ending point in 2011 was selected to combine the generated employment biographies of the register data with relevant information from the survey data at baseline (2011). Therefore, annual individual information regarding the employment status was analysed over 19 years. To examine the relationship between employment biographies and intended early retirement (H1), a chi-square test was first conducted (Table 2). Then, an ordered logit regression model (OLR) was applied (Supplementary Table 3). The distribution of socio-demographic and socio-economic variables among employment biographies is displayed in Supplementary Table 2. Analyses were performed using the statistic software IBM SPSS 25. The sequence analysis was performed in R with the package TraMineR.

#### Path analysis

A cross-lagged path analysis was applied to investigate the longitudinal association between work stress and early retirement intentions stratified by the generated employment biographies. In addition, self-rated health was used as mediator variable between work stress and early retirement intentions. The path analysis was statistically controlled for socio-demographic and socio-economic characteristics. A first model (Model 1) was constructed with direct paths of work stress on intended early retirement (H2). Then, a second model (Model 2) was applied, defined as the final model, with direct and indirect effects of work stress on early retirement intentions (Fig. 4). The final model included possible mediating effects of health by measuring the indirect effects as hypothesised (H3). To investigate causal association between work stress and health, a cross-lagged longitudinal design was constructed with data of both variables at baseline and follow-up (Reinecke 2014). Additionally, the cross-lagged approach also provides information about reversed causality (effect from health *t*_0_ to ERI *t*_1_).

Due to non-normal distributed data, the ‘asymptotic parameter-free’ estimation was calculated, and bootstrapping performed with 95%-confidence intervals based on 1000 samples (Byrne 2016). To compare direct and indirect effects, standardised regression coefficients for the paths were calculated. The indirect effects were estimated with the products of the standardised regression coefficients. In addition, in Table 5, the fit was assessed through the ‘adjusted goodness of fit index’ (AGFI), the ‘comparative fit index’ (CFI) and the ‘root mean square error of approximation’ (RMSEA). Good model fit was approved with AGFI ≥ 0.90, CFI ≥ 0.90 and RMSEA ≤ 0.05 (Byrne 2016; Hu and Bentler 1999; Hox 2010). Missing data were processed by using the imputing method of Full Information Maximum Likelihood. The path analysis was computed using AMOS 25.

## Results

### Results of the sequence analysis

**Fig. 2:**
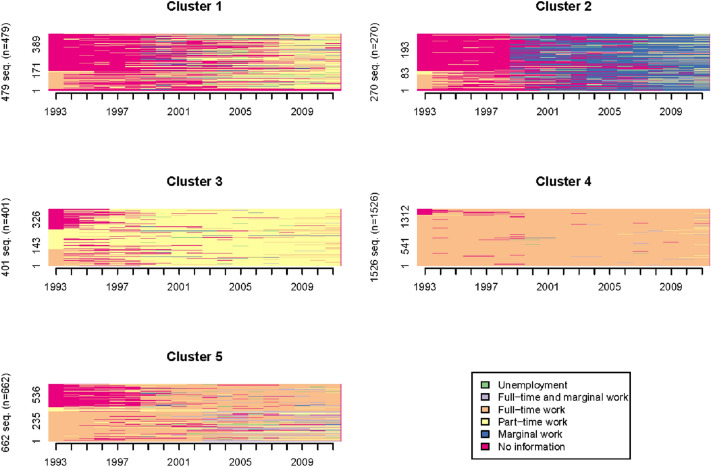
Sequence index plots of the generated clusters (*N* = 3338)

**Fig. 3:**
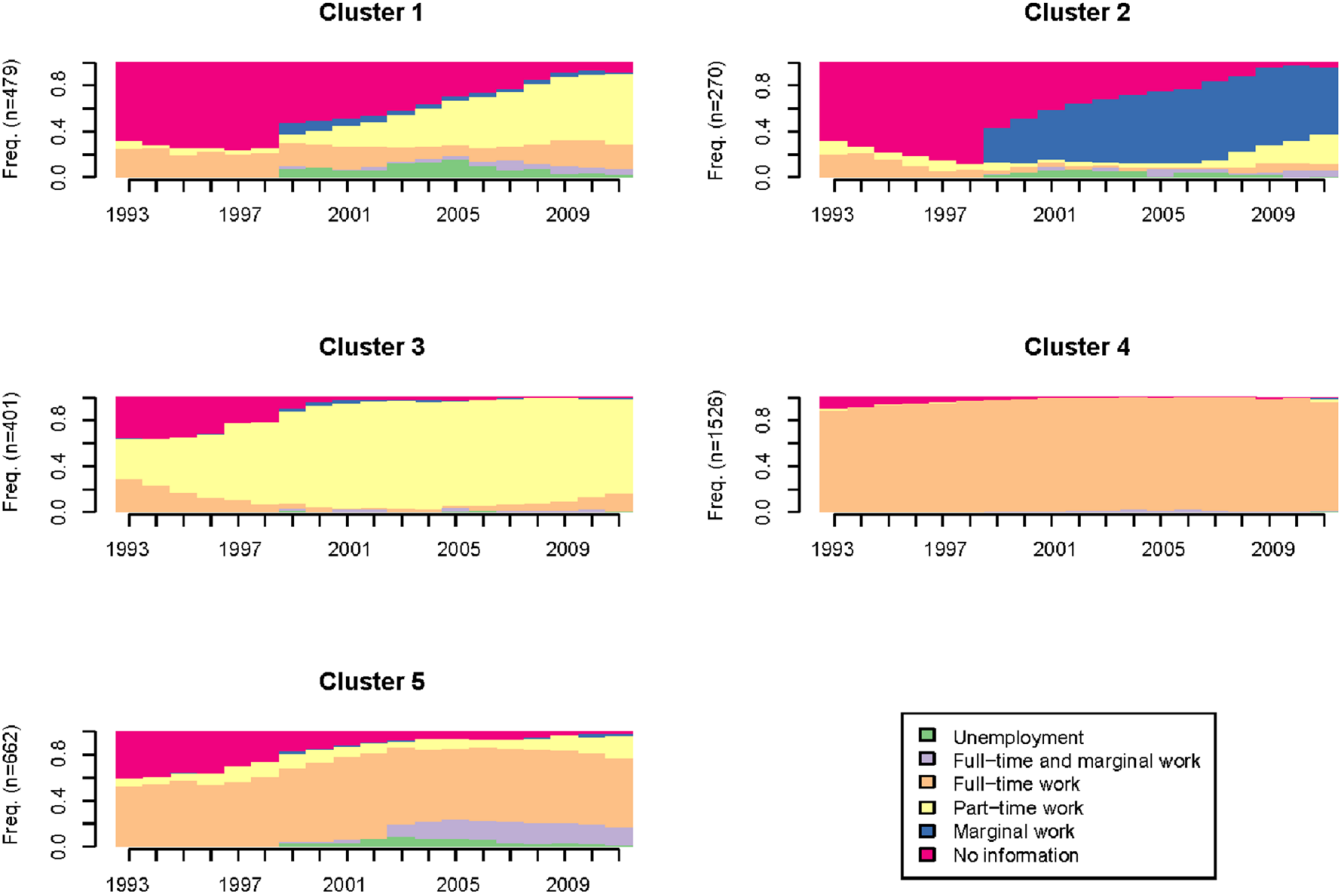
Chronograms of the generated clusters (*N* = 3338)

Five distinct clusters could be identified. Sequence index plots are displayed in Fig. 2 and render individual sequences on horizontal line segments—read from left to right. The change of colour is interpreted as a change of employment status. The so-called ‘state distribution plots’ or chronograms demonstrate the state frequencies for each year (Fig. 3). In contrast to sequence index plots, chronograms describe compositional changes of a cluster over time and do not contain information about individual sequences (Vanhoutte et al. 2018; Gabadinho et al. 2011).

The clusters are specified based on the most common employment status during our observation period (Table 1). Cluster 1 is labelled as ‘no information’, cluster 2 as ‘marginal work’, cluster 3 as ‘part-time work’, cluster 4 as ‘full-time work’ and cluster 5 as ‘full-time and marginal work’. Additionally, in Table 1, the employment biographies are classified into adverse and favourable. We defined adverse employment biographies by discontinuous and disadvantaged employment with low wages and high job insecurity. In contrast, favourable employment biographies are characterised by full-time employment with only few unemployment and few part-time periods. Therefore, clusters including mostly marginal or part-time work are defined as adverse employment biographies (cluster 1, 2 and 3). Clusters with primarily full-time jobs (cluster 4 and 5) are defined as favourable employment biographies.

**Table 1:** Employment biographies classification

Cluster	Dominant status over the years	Label	Classification
1	NI: 43.7%; P: 26.2%	No information	Adverse
2	NI: 42.1%; M: 38.6%	Marginal work	Adverse
3	P: 77.7%	Part-time work	Adverse
4	F: 95.9%	Full-time work	Favourable
5	FM: 61.9%; P: 10.1%	Full-time and marginal work	Favourable

*NI* no information, *P* part-time work, *M* marginal work, *F* full-time work, *FM* full-time and marginal work

The socio-demographic and socio-economic characteristics among clusters are displayed in Supplementary Table 2. The employment biographies differed significantly regarding sex, age, education, occupational status, and income.

In Table 2, employment biographies and early retirement intentions are compared in a crosstab. Results of the chi-square test showed that employment biographies differed significantly regarding intended retirement age. The highest percentage of people wanting to retire early could be identified in the employment biography ‘part-time work’; the next highest was found in ‘full-time work’. People working predominantly in marginal employment had the lowest early retirement intentions. Intended early retirement could be found in both, adverse and favourable, employment biographies. The results of the OLR showed associations between employment biographies and intended early retirement (Supplementary Table 3). The adverse employment biography ‘marginal work’ had the intention to retire later than the ‘full-time work’. No significant association with intended early retirement was found for the other employment biographies. Therefore, hypothesis 1 cannot be accepted.

**Table 2:** Comparison of employment biographies and intended retirement age in absolute numbers^a^

Intended retirement age						
Employment biographies	50–54 years	55–59 years	60–64 years	65–67 years	Beyond the statutory retirement age	Total
No information	16 (3.3%)	142 (29.7%)	231 (48.2%)	49 (10.2%)	41 (8.6%)	479 (100%)
Marginal work	7 (2.6%)	68 (25.2%)	128 (47.4%)	36 (13.3%)	31 (11.5%)	270 (100%)
Part-time work	11 (2.7%)	122 (30.4%)	235 (58.6%)	26 (6.5%)	7 (1.8%)	401 (100%)
Full-time work	54 (3.5%)	404 (26.5%)	893 (58.5%)	125 (8.2%)	50 (3.3%)	1,526 (100%)
Full-time and marginal work	23 (3.5%)	180 (27.2%)	350 (52.9%)	71 (10.7%)	38 (5.7%)	662 (100%)
Total	111 (3.3%)	916 (27.5%)	1,837 (55.0%)	307 (9.2%)	167 (5.0%)	3,338 (100%)

^a^Percentage by row

Chi-square test: *χ*^2^ (16) = 81.25; *p* < 0.001

### Results of the path analysis

The path analysis without mediators (Model 1) is displayed in detail in supplementary Figure 1. The results of direct effects of ERI on early retirement intentions, stratified by employment biographies, are shown in Table 3. Increased work stress at baseline was associated with earlier retirement intentions at follow-up. This association was found to be significant in each employment biography, except ‘full-time and marginal work’. Respectively, hypothesis 2 can be partially confirmed.

**Table 3:** Direct effects^a^ of work stress (ERI *t*_0_) on early retirement intentions (*t*_1_); path analysis without health as mediator

Employment biographies					
Path	No information	Marginal work	Part-time work	Full-time work	Full-time and marginal work
ERI	− 0.114*	− 0.170**	− 0.272*	− 0.109***	− 0.045

^a^Standardised regression weights

*p < 0.05. **p < 0.01. ***p < 0.001

**Fig. 4:**
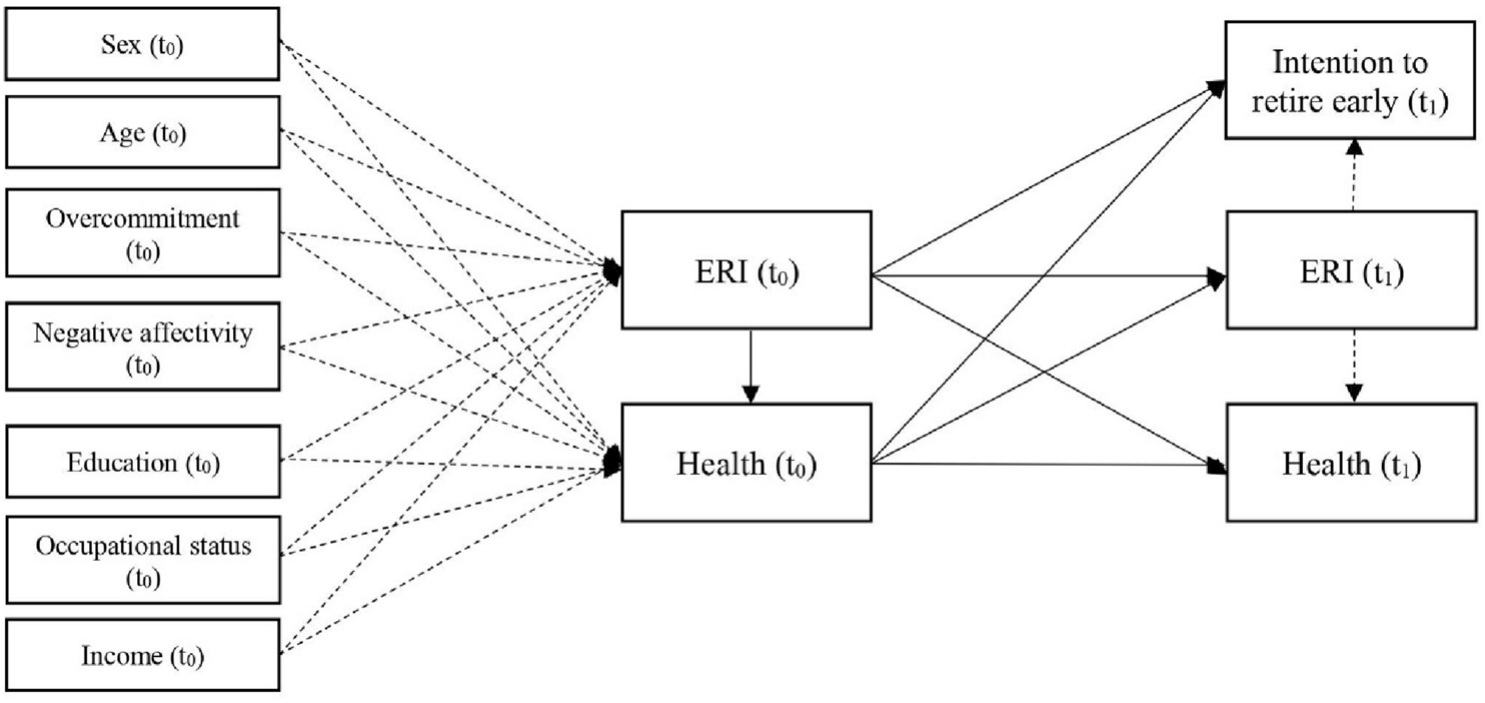
Final path analysis with cross-lagged approach stratified by employment biographies

The final path analysis model with cross-lagged approach and health as mediator is displayed in Fig. 4. In Table 4, the results of the final path model (Model 2) are presented with standardised regression weights for each employment biography. The full path models for each employment biography are displayed in Supplementary Figure 2 to 6. Direct effects of ERI (*t*_0_) on intended early retirement (*t*_1_) were found. Increased work stress at baseline was associated with early retirement intentions at follow-up for the clusters: ‘no information’, ‘marginal work’ and ‘full-time work’. Significant indirect effects were found for the employment biographies: ‘no information’, ‘part-time work’ and ‘full-time work’. Higher work stress was associated with poorer health, likewise poorer health with early retirement intentions. Therefore, we can partially approve hypothesis 3.

**Table 4:** Direct and indirect effects of work stress (ERI *t*_0_) on intended early retirement (*t*_1_) in the full path model; health^b^ (*t*_0_) as mediator

Path	No information	Marginal work	Part-time work	Full-time work	Full-time and marginal work
	*Direct effects*^a^				
ERI $\to$ early retirement intentions	− 0.094*	− 0.149***	− 0.063	− 0.084**	− 0.048
ERI $\to$ health	0.110*	− 0.016	0.164**	0.168***	0.202***
Health $\to$ early retirement intentions	− 0.119*	− 0.191***	− 0.092*	− 0.096***	− 0.078
	*Indirect effects*^a^				
ERI $\to$ health $\to$ early retirement intentions	− 0.013*	0.003	− 0.015*	− 0.016*	0.016

^a^Standardised regression weights

^b^Higher values indicate poorer health

*p < 0.05. **p < 0.01. ***p < 0.001

A good model fit was found for all clusters (Table 5). The estimators of CFI (0.879) and RMSEA (0.060) were at the threshold of goodness of fit only for the employment biography ‘marginal work’.

**Table 5:** Goodness of fit of the final path model with health as mediator (Model 2)

	No information	Marginal work	Part-time work	Full-time work	Full-time and marginal work
AGFI^a^	0.953	0.957	0.988	1.000	0.997
CFI^b^	0.948	0.879	0.987	0.993	0.965
RMSEA^c^	0.044	0.060	0.020	0.017	0.038

^a^*AGFI* Adjusted Goodness of Fit Index

^b^*CFI* Comparative Fit Index

^c^*RMSEA* Root Mean Square Error of Approximation

## Discussion

This study illustrates how psychosocial factors (work stress) and health can influence early retirement intentions among different employment histories. We were able to distinguish different employment biographies and their association with further labour market participation, which underlines the importance of a life course perspective. Our findings suggest that specific employment biographies interact with work stress and with poor health regarding intended early retirement. Employment biographies were defined by specific work contract characteristics (such as full-time or part-time) and by employment characteristics (such as employed or unemployed), which are correlated with certain socio-demographic and socio-economic characteristics (Supplementary Table 2). Adverse employment biographies showed a high proportion of women, while favourable ones were characterised by mostly men or equally women and men. The favourable employment biographies had higher income and higher occupational status compared to the adverse ones. In order to control for these variations in employment biographies, the path analysis was adjusted for these factors. Additionally, results of the path analysis showed that the effects differ more strongly between the employment biographies when it was not controlled for occupational status and income. In particular, the effects observed in adverse employment biographies were mostly reduced (not displayed in detail).

Our findings of the chi-square test demonstrated that early retirement intentions differ dependent on previous labour market involvement—the employment biographies. The descriptive results demonstrated that the highest intention to retire early could be found in the adverse employment biography ‘part-time work’. In addition, the findings showed that favourable employment biographies were also associated with early retirement intentions. However, the results of the OLR showed that people working predominantly in marginal employment want to retire later compared to full-time workers. Therefore, H_1_ cannot be accepted. It appears that employment types itself were not influencing early retirement intentions but a complex combination of other factors. Previous research showed that retirement decisions can be influenced by various push and pull factors. For example, a partner that is already retired can represent a pull factor for early retirement (Shultz et al. 1998). However, in our study, there was no significant correlation found between the employment status of the partner and early retirement intentions (spearman rank correlation coefficient (*r*_s_) = 0.018; *p* > 0.05). Furthermore, financial factors represent a relevant push factor of retirement decisions (Shultz et al. 1998). Financial concerns can influence the intention to retire early (Du Prel et al. 2019). In our study, a weak but significant association between lack of financial scope of and early retirement intentions could be identified (*r*_s_ = 0.05; *p* < 0.05). Regarding our first hypothesis, adverse employment biographies presumably intend to retire later due to their financial difficulties, and therefore, a clear direction could not be observed in our study. The results of the path analysis showed that high work stress can be associated with early retirement intentions and therefore confirm early findings (Du Prel et al. 2019; Wahrendorf et al. 2013; Siegrist et al. 2007). This longitudinal association was found in all employment biographies except for the employment biography ‘full-time and marginal work’. Unlike our assumptions, besides employment biographies with predominantly adverse working conditions, full-time workers with high work-related stress also have early retirement intentions. This result surprises and indicates that long exposure time of full-time workers can lead to high work stress and thus early retirement intentions. Respectively, hypothesis 2 can be partially confirmed through the significant association found in both, adverse and favourable employment biographies. Furthermore, the path analysis revealed a mediation through health on the association between work stress and early retirement intentions in the three employment biographies: ‘no information’, ‘part-time work’ and ‘full-time work’. Higher work stress was associated with poorer health, and poorer health with early retirement intentions. No indirect effect of health was found for ‘marginal work’ and ‘full-time and marginal work’. Therefore, H_3_ can be partially accepted. In addition, the estimates suggested that poorer health at baseline was associated with earlier retirement intentions at follow-up. In contrast to recent research, the finding of our study showed a clear association between health and intended early retirement (Du Prel et al. 2019). Therefore, our results are in line with other previous research (von Bonsdorff et al. 2010; Harkonmäki 2007; Siegrist et al. 2007; Elovainio et al. 2005). Moreover, after controlling for health, direct effects of work stress on early retirement intentions were still significant for some employment biographies (‘no information’, ‘marginal work’ and ‘full-time work’). This indicates that health and work stress could irrespectively be linked to early retirement intentions, as also observed in earlier research (Siegrist et al. 2007). However, only with health as mediator was an association between work stress and early retirement intentions observed for those working predominantly part-time. This result indicates that especially for part-time workers, health instead of work stress can be defined as a more relevant influence on early retirement intentions. Earlier research showed that push and pull factors can affect workers in different ways (Shultz et al. 1998). Poor health is considered one major reason for early retirement (Harkonmäki 2007; Elovainio et al. 2005; von Bonsdorff et al. 2010; Siegrist et al. 2007). However, it seems reasonable that for people with adverse employment biographies, health and financial aspects have a diametric effect regarding the intention to retire earlier. Therefore, we cannot exclude that adverse employment biographies will have to work longer in the future due to financial concerns, despite possible health restrictions, and will postpone their early retirement intentions.

In Germany and many other European countries, social security systems will face more pressure from the ageing workforce and the upcoming retirement of large birth cohorts. As a result, many European governments try to prevent early exits from the labour market through particular pension reforms (European Commission 2020). Therefore, we think that the results of our study could be interesting and helpful for other countries with similar issues. Implementing strategies to decrease work stress and promote health among workers are important to reduce early retirement intentions and provide a strong workforce for as long as possible.

### Strengths and limitations

There are limitations regarding our data that should be mentioned. First, in the lidA cohort study civil servants, the self-employed and freelancers are excluded, and therefore, generalizability of our findings is limited. Secondly, the IEB data contain limitations that should be mentioned. Due to the composition, long-term sick people could not be included; only those who were incapable of work for less than 42 days were considered. Another restriction of the IEB data refers to information on marginal employment and unemployment, which was not available before 1999. Hence, considering Dannefer’s theory of cumulative advantage/disadvantage, the effect of adverse clusters could be underestimated in this study. Furthermore, giving the study attrition rate between *t*_0_ and *t*_1_, we checked for possible selection bias. We could not observe a selection bias. Third, other limitations consider the study methodology. The sequence analysis only considered employment status information that lasted more than 6 months. Consequently, short-term work was not recorded, and thus, the occurrence may have been underestimated. Furthermore, the generated employment status ‘no information’ included different groups of people. However, the composition of the register data did not allow further distinction. Therefore, it is possible that the diversity of the employment status ‘no information’ may be underestimated. Fourth, a limitation of the path model is referring to the measurement of the mediating variable health, which was measured at the same time as work stress. Yet, the relationship between health (*t*_0_) and work stress (*t*_0_) was found to be weak (*r*_s_ = 0.273; *p* < 0.05). Moreover, the data lag of three years (2011 vs. 2014) could occur as a limitation. In the model of effort-reward imbalance, work stress is considered to be chronic stress although it is expected to be sensitive to changes in the psychosocial work environment (Siegrist et al. 2004). However, we did not formulate explicit assumptions how changes of ERI and of health over time should affect the hypothesised associations. Therefore, lagged effects are not expected to bias our results. Finally, we defined the employment biographies regarding different employment statuses over time, which are correlated with certain socio-economic characteristics. The separation between adverse and favourable employment biographies in this study represents only one possibility of classification.

There are several strengths of this study. The first strength refers to the high representativeness of this study for German employees subject to social security contributions of the two birth cohorts (1959 and 1965). Second, the IEB data itself represent a major strength of our study. It contains highly comprehensive and reliable information gathered from employers’ yearly reports submitted to the social security authorities (Hasselhorn et al. 2014). Moreover, compared to previous studies using retrospective data, our data are free from possible recall bias. Third, the data could be analysed over a period with an appropriate life course approach, as opposed to the mostly cross-sectional approach as in earlier studies. Regarding the relationship between work stress and health, reverse causality could not be eliminated by the cross-lagged approach, but statistically controlled to a certain extent. No significant effect of health *t*_0_ on ERI *t*_1_ was found (Supplementary Table 4).

## Conclusion

The growth of precarious employment in recent years is shaping employment biographies today. A conclusion regarding the influence of previous labour market participation and psychosocial factors on early retirement intentions could not be drawn from earlier research. The present study investigates and highlights the importance of the life course approach regarding the influences on retirement decisions. Our results demonstrate that high work stress influences early retirement intentions among both adverse and favourable employment biographies. Moreover, health can impact this association. Therefore, when considering the ageing society and preventing early retirement intentions, working conditions such as reducing work stress should be prioritised along with focussing on health-promoting interventions in the labour market. Additionally, earlier studies showed a distinction between retirement intentions and behaviours. Therefore, our results suggest that further research could build on our findings and focus on the influence of psychosocial factors and health on actual retirement.

## Supplementary Information

Below is the link to the electronic supplementary material.

Supplementary file1 (DOCX 399 KB)

## Data Availability

The research data contain social security information. Due to legal regulations in Germany, it is not permitted to share data with social security information. Hence, the research data are confidential.
