# Supplementary material for: Early retirement intentions: the impact of employment biographies, work stress and health among a baby-boomer generation
Source: Eur J Ageing. 2022 Sep 30;19(4):1479–91. doi: 10.1007/s10433-022-00731-0 (PMC9729676; doi:10.1007/s10433-022-00731-0)
Supplement: Supplementary file 1 — Supplementary file1 (DOCX 399 KB) [file 10433_2022_731_MOESM1_ESM.docx]

Supplementary Material

**Supplementary Table 1** Definition of the variable ‘education’ using combined information about education and vocational training

|  | |  | Education | | | | | |
| --- | --- | --- | --- | --- | --- | --- | --- | --- |
|  |  |  | No qualification | Lower secondary school (8/9 years) | Secondary school (10 years) | Technical college (12/13 years) | Academic secondary school (12/13 years) | Other qualification |
| Vocational training | No qualification |  | 1 | 1 | 1 | 2 | 2 | 1 |
|  | Vocational school – company |  | 1 | 1 | 2 | 2 | 2 | 1 |
|  | Vocational school – college |  | 1 | 1 | 2 | 2 | 2 | 1 |
|  | Specialized secondary school |  | - | 2 | 2 | 2 | 2 | 2 |
|  | University of applied sciences |  | - | - | 3 | 3 | 3 | 3 |
|  | University |  | - | - | 3 | 3 | 3 | 3 |
|  | Other qualification |  | - | 1 | 2 | 2 | 3 | 1 |

**Supplementary Table 2** Distribution of socio-demographic and socio-economic indicators in employment biographies (in %) and results of the chi-square test to analyse variation among employment biographies

|  | Employment biographies | | | | |  |
| --- | --- | --- | --- | --- | --- | --- |
| Variable (t_0_) | No information  (n=479) | Marginal work  (n=270) | Part-time work (n=401) | Full-time work (n=1,526) | Full-time and marginal work (n=662) | p^a^ |
| Sex |  |  |  |  |  | *** |
| Male | 16.1 | 8.9 | 4.0 | 73.9 | 46.4 |  |
| Female | 83.9 | 91.1 | 96.0 | 26.1 | 53.6 |  |
| Year of birth |  |  |  |  |  | *** |
| 1959 | 33.8 | 39.3 | 58.9 | 48.2 | 38.2 |  |
| 1965 | 66.2 | 60.7 | 41.1 | 51.8 | 61.8 |  |
| Education |  |  |  |  |  | *** |
| High | 26.7 | 18.9 | 15.2 | 23.0 | 23.9 |  |
| Intermediate | 58.2 | 54.4 | 66.1 | 53.5 | 55.7 |  |
| Low | 15.0 | 26.7 | 18.7 | 23.5 | 20.4 |  |
| Occupational status |  |  |  |  |  | *** |
| Professionals | 3.6 | 1.5 | 1.2 | 5.4 | 4.2 |  |
| Middle management   workers | 31.3 | 17.1 | 35.9 | 46.2 | 38.7 |  |
| Skilled workers | 43.4 | 40.7 | 47.4 | 38.1 | 36.1 |  |
| Unskilled workers | 21.7 | 40.7 | 15.5 | 10.3 | 21.0 |  |
| Income |  |  |  |  |  | *** |
| High | 4.8 | 2.6 | 1.2 | 20.5 | 9.8 |  |
| Middle high | 8.6 | 3.3 | 8.0 | 33.8 | 20.1 |  |
| Middle low | 45.3 | 15.9 | 57.4 | 42.3 | 51.8 |  |
| Low | 41.3 | 78.2 | 33.4 | 3.4 | 18.3 |  |

^a^ p-value of Pearson chi-square test.
* = p<0.05. ** = p<0.01. *** = p<0.001.

**Supplementary Table 3** Results of the ordered logit regression model estimating associations between intended early retirement (intended retirement age) and employment biographies

|  | Model A^1^ | Model B^2^ | Model C^3^ |
| --- | --- | --- | --- |
| Variables | Estimates | | |
| Intended retirement age  (ref. beyond the statutory retirement age |  |  |  |
| 50-54 years | -3.319*** | -3.821*** | -4.201*** |
| 55-59 years | -0.757*** | -1.235*** | -1.609*** |
| 60-64 years | 1.847*** | 1.410*** | 1.050*** |
| 65-67 years | 2.998*** | 2.582*** | 2.227*** |
| Employment biography  (ref. full-time) |  |  |  |
| No information | 0.097 | 0.062 | 0.060 |
| Marginal work | 0.480*** | 0.360** | 0.360** |
| Part-time work | -0.161 | -0.145 | -0.149 |
| Full-time and marginal work | 0.112 | 0.128 | 0.131 |
| ERI (t_0_) | - | -0.809*** | -0.638*** |
| Health (t_0_) ^a^ | - | - | -0.189*** |

* = p<0.05. ** = p<0.01. *** = p<0.001.
^1^ Model A includes only employment biographies as a factor.
^2^ Model B additionally controls for work stress at baseline.
^3^ Model C controls for both work stress and health at baseline.
^a^ Higher values indicate poorer health.


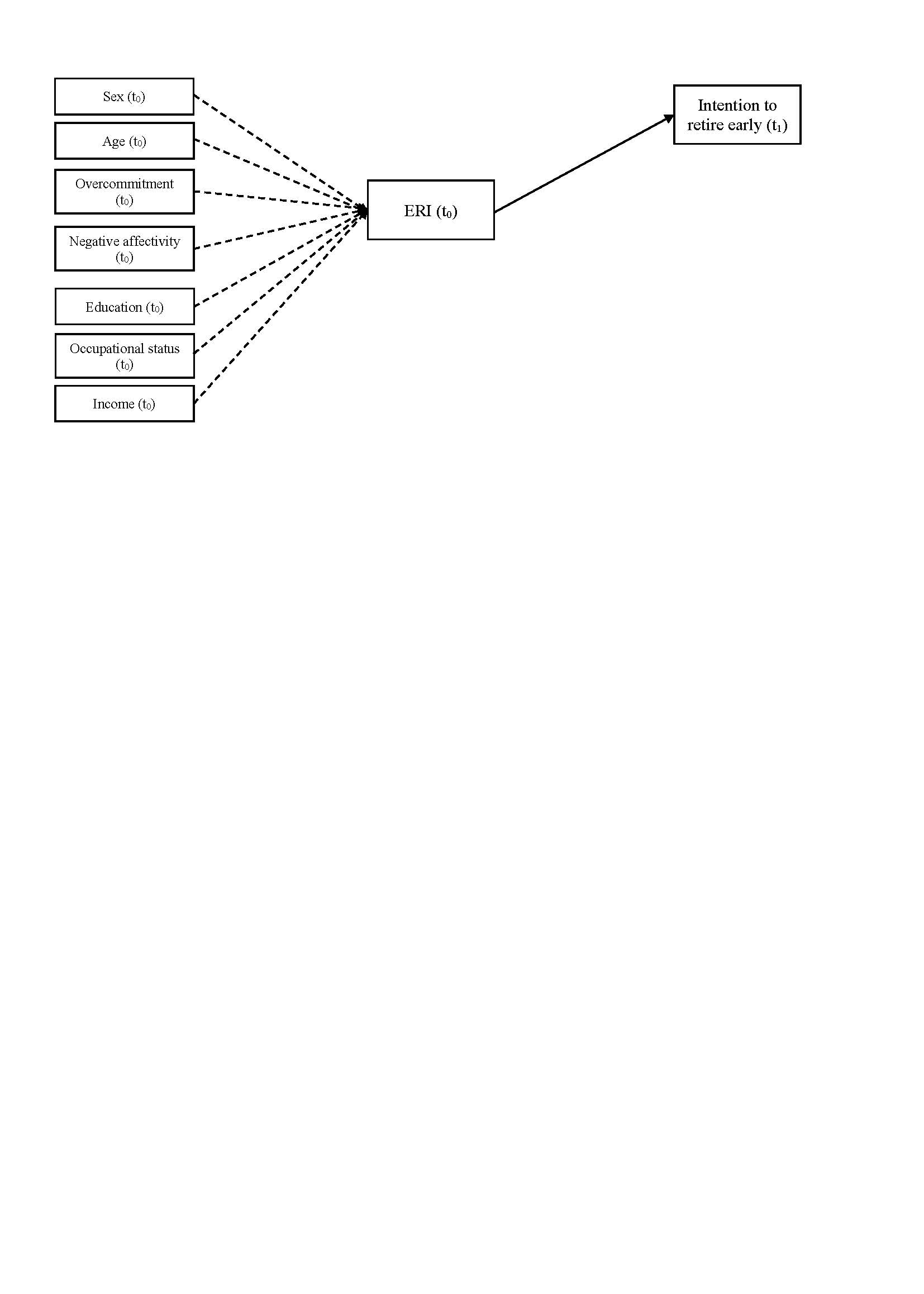


**Supplementary Figure 1** Path analysis (Model 1) without health as mediator stratified by employment biographies

ERI (t_0_)

Health (t_0_)

Intention to retire early (t_1_)

ERI (t_1_)

Health (t_1_)

Age (t_0_)

Sex (t_0_)

-0.017

-0.014

0.043

-0.101*

-0.119*

-0.094*

Overcommitment (t_0_)

0.507***

0.634***

0.010

0.131*

Negative affectivity (t_0_)

0.206***

0.004

0.289***

0.110*

0.076

-0.147**

Education (t_0_)

0.111*

0.520***

0.096*

-0.086*

Occupational status (t_0_)

0.003*

Income (t_0_)

0.023

**Supplementary Figure 2** Standardised regression weights of the full path analysis (Model 2) with health as mediator – employment biography: ‘No information’ (cluster 1)

ERI (t_0_)

Health (t_0_)

Intention to retire early (t_1_)

ERI (t_1_)

Health (t_1_)

Age (t_0_)

Sex (t_0_)

0.032

-0.065

-0.095

-0.149***

-0.191***

Overcommitment (t_0_)

0.148***

0.479***

0.767***

0.083

0.277***

Negative affectivity (t_0_)

0.228***

0.005

0.295**

-0.016

0.078

-0.143

Education (t_0_)

0.074

0.455***

0.120*

0.037

Occupational status (t_0_)

0.052

Income (t_0_)

-0.065

**Supplementary Figure 3**  Standardised regression weights of the full path analysis (Model 2) with health as mediator – employment biography: ‘Marginal work’: (cluster 2)

ERI (t_0_)

Health (t_0_)

Intention to retire early (t_1_)

ERI (t_1_)

Health (t_1_)

Age (t_0_)

Sex (t_0_)

-0.016

0.011

-0.063

0.052

-0.053

-0.092*

Overcommitment (t_0_)

0.519***

0.620***

0.046

0.139*

Negative affectivity (t_0_)

-0.032

0.120*

0.312***

0.164**

-0.009

0.056

Education (t_0_)

0.121*

0.478***

-0.033

0.038

Occupational status (t_0_)

-0.011

Income (t_0_)

0.000

**Supplementary Figure 4** Standardised regression weights of the full path analysis (Model 2) with health as mediator – employment biography: ‘Part-time work’ (cluster 3)

ERI (t_0_)

Health (t_0_)

Intention to retire early (t_1_)

ERI (t_1_)

Health (t_1_)

Age (t_0_)

Sex (t_0_)

-0.023

-0.007

0.042*

-0.096***

-0.084**

Overcommitment (t_0_)

-0.099***

0.519***

0.787***

0.125***

Negative affectivity (t_0_)

0.173***

0.084***

0.045

0.168**

0.296***

0.051*

-0,104**

Education (t_0_)

0.120***

0.451***

0.087**

0.038

Occupational status (t_0_)

0.046

Income (t_0_)

0.096***

**Supplementary Figure 5** Standardised regression weights of the full path analysis (Model 2) with health as mediator – employment biography: ‘Full-time work’ (cluster 4)

-0.069

ERI (t_0_)

Health (t_0_)

Intention to retire early (t_1_)

ERI (t_1_)

Health (t_1_)

Age (t_0_)

Sex (t_0_)

-0.046

-0.001

-0.029

-0.078

-0.048

Overcommitment (t_0_)

0.497***

0.541***

0.181***

Negative affectivity (t_0_)

0.069

0.054

0.113**

0.202***

0.296***

0.086*

-0.100**

Education (t_0_)

0.138***

0.492***

0.087**

0.069

Occupational status (t_0_)

0.089*

Income (t_0_)

0.032

**Supplementary Figure 6**  Standardised regression weights of the full path analysis (Model 2) with health as mediator – employment biography: ‘Full-time and marginal work’ (cluster 5)

**Supplementary Table 4** Direct effects^a^ testing reversed causality between work stress (ERI) and health

| Path | No information | Marginal work | Part-time work | Full-time work | Full-time and marginal work |
| --- | --- | --- | --- | --- | --- |
| ERI (t_0_) 🡪 health (t_1_) | -0.147** | -0.143 | -0.032 | -0.104** | -0.100** |
| Health (t_0_) 🡪 ERI (t_1_) | 0.004 | 0.005 | 0.056 | 0.045 | 0.054 |

* = p<0.05. ** = p<0.01. *** = p<0.001. ^a^ Standardised regression weights.
t_0_ = baseline in 2011. t_1_ = follow-up in 2014.
